# Visibility of esophageal squamous cell carcinoma under iodine staining on texture and color enhancement imaging

**DOI:** 10.1002/deo2.370

**Published:** 2024-05-08

**Authors:** Tsunetaka Kato, Takuto Hikichi, Jun Nakamura, Minami Hashimoto, Ryoichiro Kobashi, Takumi Yanagita, Tadayuki Takagi, Rei Suzuki, Mitsuru Sugimoto, Hiroyuki Asama, Yuki Sato, Yasuo Shioya, Masao Kobayakawa, Hiromasa Ohira

**Affiliations:** ^1^ Department of Endoscopy Fukushima Medical University Hospital Fukushima Japan; ^2^ Department of Gastroenterology Fukushima Medical University School of Medicine Fukushima Japan; ^3^ Medical Research Center Fukushima Medical University Fukushima Japan

**Keywords:** color difference, endoscopic diagnosis, esophageal squamous cell carcinoma, iodine staining, texture and color enhancement imaging

## Abstract

**Objective:**

Iodine staining on white light imaging (WLI) is the gold standard for detecting and demarcating esophageal squamous cell carcinoma (ESCC). We examined the effects of texture and color enhancement imaging (TXI) on improving the endoscopic visibility of ESCC under iodine staining.

**Methods:**

Twenty ESCC lesions that underwent endoscopic submucosal dissection were retrospectively included. The color difference between ESCC and the surrounding mucosa (*ΔE*e) on WLI, TXI, and narrow‐band imaging was assessed, and *ΔE*e under 1% iodine staining on WLI and TXI. Furthermore, the visibility grade determined by endoscopists was evaluated on each imaging.

**Result:**

The median *ΔE*e was greater on TXI than on WLI (14.53 vs. 10.71, respectively; *p* < 0.005). Moreover, the median *ΔE*e on TXI under iodine staining was greater than the median *ΔE*e on TXI and narrow‐band imaging (39.20 vs. 14.53 vs. 16.42, respectively; *p* < 0.005 for both). A positive correlation in *ΔE*e under iodine staining was found between TXI and WLI (correlation coefficient = 0.61, *p* < 0.01). Moreover, *ΔE*e under iodine staining on TXI in each lesion was greater than the corresponding *ΔE*e on WLI. The visibility grade assessed by endoscopists on TXI was also significantly greater than that on WLI under iodine staining (*p* < 0.01).

**Conclusions:**

The visibility of ESCC after iodine staining was greater on TXI than on WLI.

## INTRODUCTION

Among esophageal cancers, squamous cell carcinoma (SCC) accounts for 90% in East Asia.^1^, ^2^ Esophageal SCC (ESCC) at cStage 0 is indicated for endoscopic resection, and sufficient outcomes have been achieved with endoscopic resection.^3^, ^4^ Iodine staining is the gold standard for the detection and border demarcation of cStage 0 ESCC.^5^, ^6^, ^7^, ^8^, ^9^, ^10^, ^11^ However, iodine staining of the esophageal mucosa may cause adverse events, such as chest pain, laryngitis, pneumonia, and gastric mucosal injury.^12^, ^13^, ^14^, ^15^ For patients at high risk for ESCC, endoscopic examination is often performed under sedation or through image‐enhanced endoscopy, such as narrow‐band imaging (NBI), to avoid the use of iodine staining. Although iodine staining has been used at a concentration of approximately 1% in recent years, adverse events still occur frequently, making the use of a lower concentration of iodine staining potentially desirable.^16^

Recently, a new image‐enhanced endoscopy technique, texture and color enhancement imaging (TXI), has been developed.^17^ TXI is designed to emphasize the three image elements of white light imaging (WLI): texture, color, and brightness. Furthermore, TXI has two modes. TXI has been reported to improve the visibility of gastrointestinal lesions by enhancing mucosal structures in real‐time during endoscopic observation.^17^ The effectiveness of TXI compared with WLI in improving the visibility of gastric and colorectal cancer has been reported.^18^, ^19^, ^20^, ^21^, ^22^, ^23^ Improved visibility on TXI for ESCC has also been reported^24^; however, the effects of TXI when iodine staining is used for ESCC have not been evaluated. Here, we preliminary examined the effects of TXI on improving the endoscopic visibility of ESCC under 1% iodine staining.

## METHODS

### Study design and patients

We retrospectively collected data on patients with ESCC who underwent endoscopic submucosal dissection (ESD) at Fukushima Medical University Hospital from March 2022 to September 2022. Patients who did not undergo endoscopic examinations with EVIS X1 (Olympus Medical Systems Corp.) were excluded. We targeted a minimum of 20 cases as a pilot study.

This study was conducted according to the Guidelines of the Declaration of Helsinki and was approved by the Ethics Committee of Fukushima Medical University (approval number: 2020–146).

### Endoscopic imaging and ESD for ESCC in the clinical setting

According to the Japanese Guidelines for the Treatment of Esophageal Cancer,^25^ ESCCs that appeared to be within the mucosa, without lymph node or distant metastasis, were indicated for ESD. If the patients did not have an allergy to iodine, precise observation with iodine staining was performed in all cases just before ESD. Endoscopic observation and ESD procedures were performed by five expert endoscopists who were board‐certified fellows of the Japan Gastroenterological Endoscopy Society.

**FIGURE 1:**
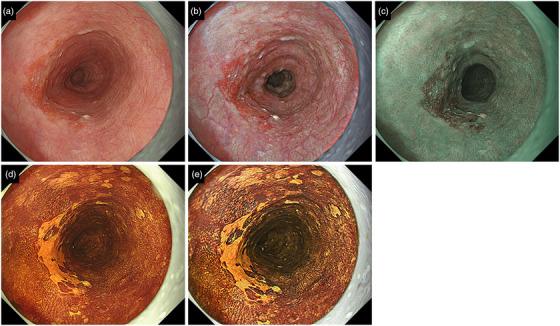
A representative case of esophageal squamous cell carcinoma. WLI showed a red depressed lesion with less than 1/2 circumference in the middle of the thoracic esophagus (a). The same lesion as Figure 1a was imaged using TXI Mode 1 (b) and NBI (c). Furthermore, WLI (d) and TXI (e) images were taken after spraying with 1% iodine solution. The histopathological findings of the ESD specimen showed SCC with a depth of the MM and a lesion length diameter of 26 mm. WLI, white light imaging; TXI, texture, and color enhancement imaging; NBI, narrow‐band imaging; ESD, endoscopic submucosal dissection; MM, muscularis mucosae; SCC, squamous cell carcinoma.

GIF‐EZ1500 or GIF‐XZ1200 (Olympus Medical Systems Corp.) with the EVIS X1 endoscope system was used. Three types of images, WLI (Figure 1a), TXI (Figure 1b), and NBI (Figure 1c), were taken, each with 4–10 frames. For TXI, only TXI Mode 1 was used. Following this, a 1% iodine solution was sprayed over the entire lesion using a catheter. Endoscopic image enhancement was set to B8 for WLI and NBI. Additionally, TXI Mode1 was set to strong enhancement. WLI and TXI images were taken within one minute after iodine staining, 4–10 frames at a time (Figures 1d,e). Therefore, the pink‐color sign was not evaluated. All endoscopic images were stored in Solemio ENDO (Olympus Medical Systems Corp.).

According to the Japanese Classification of Esophageal Cancer, the histopathological features of resected specimens, including macroscopic type, lesion diameter, lesion depth, lymphatic invasion, venous invasion, horizontal margin, and vertical margin, were evaluated.^26^

### Evaluation of color difference

The c (CIE) LAB color system recommended by the CIE in 1976 as a method for quantifying color^27^ was adopted in this study. The concept of a “color space” comprised the L*, a*, and b* axes. L* represents “lightness,” indicated by black on white (range, 0–100). a* and b* represent “chromaticity,” respectively. Green represents the negative direction of a*, whereas red represents the positive direction (range, −60–60). Blue represents the negative direction of b*, whereas yellow represents the positive direction (range, −60–60). However, because endoscopic image colors are output in the red, green, and blue (RGB) color system, L*a*b* values were calculated by converting RGB color system values to CIE LAB color system values based on the conversion formula of Mokrzycki et al.^27^ Color difference (*ΔE*) is a concept that quantifies the difference between two colors and can be expressed as the distance between two colors in the “color space.”

**FIGURE 2:**
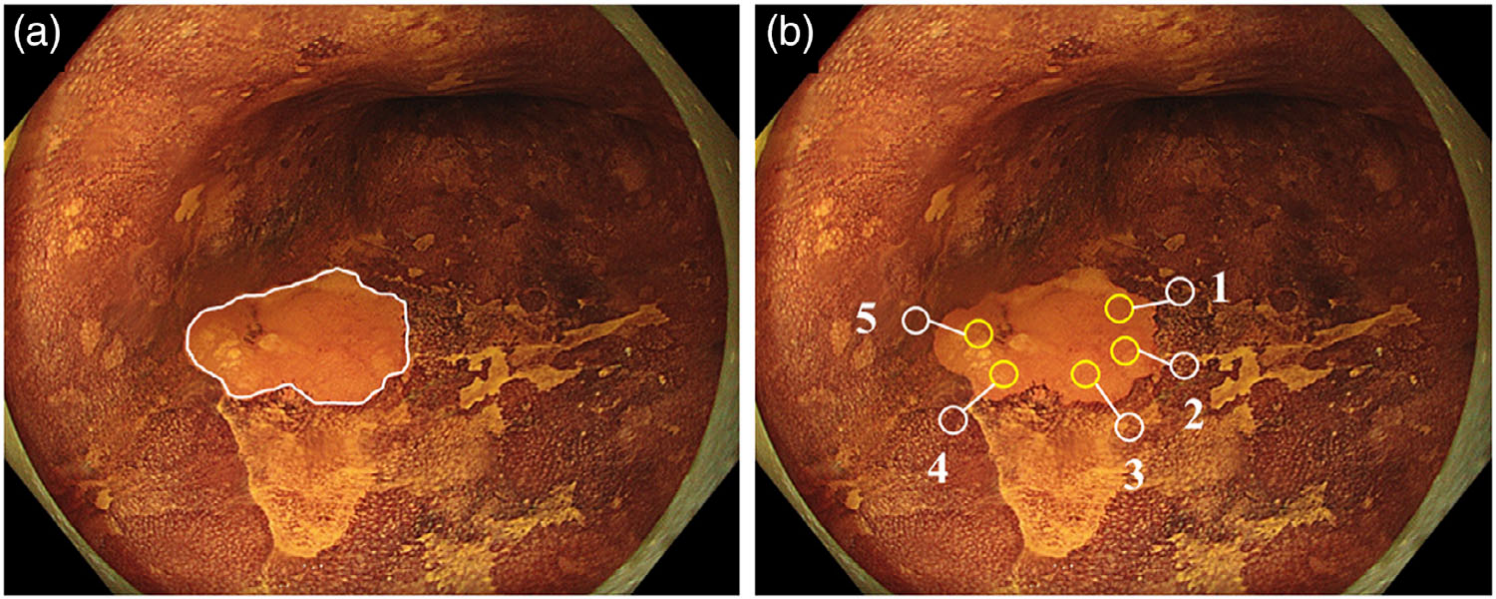
Selection of sample points for *ΔE*_e_ calculation. Based on the histopathological results of ESD, the border between the ESCC and surrounding mucosa is defined by a white line on the endoscopic image (a). Next, five sample points are randomly selected from the ESCC and surrounding mucosa, each in pairs (b). *ΔE*e, the color difference between the esophageal squamous cell carcinoma and the surrounding mucosa; ESD, endoscopic submucosal dissection; ESCC, esophageal squamous cell carcinoma.

Based on a previous theory, *ΔE* was measured from endoscopic images as follows: one endoscopist (Tsunetaka Kato) selected one image each from the endoscopic images that approximated the distance and angle between the lesion and endoscope. The images were placed on image analysis software (Adobe Photoshop Elements 12; Adobe Systems), and five sample points were randomly selected from each of the ESCC and surrounding mucosa (10 sample points in total) as a pair (Figure 2a,b). The sample point selected was within 2 mm of the borderline between the ESCC and surrounding mucosa, which was determined based on the pathology results of ESD. The RGB values of each sample point were converted to L*a*b* values using a conversion formula. Using the L*a*b* values (L*y, a* y, b* y) of the ESCC and the L*a*b* values (L*y, a* y, b* y) of the surrounding mucosa, *ΔE* between the paired ESCC and surrounding mucosa was calculated using the following formula: *ΔE* = √ (L*_x_ − L*_y_)^2^ + (a*_x_ − a*_y_)^2^ + (b*_x_ − b*_y_)^2^

The *ΔE* average at 5 points calculated here was defined as the *ΔE*e. Moreover, the difference in brightness between the ESCC and surrounding mucosa was defined as “ΔL* = L*x − L*y” and the difference in chromaticity as “Δa* = a*x − a*y” and “Δb* = b*x − b*y” were defined.

### Outcomes

Clinical data were retrospectively collected from electronic medical records and endoscopic databases. The following outcomes were assessed: *ΔEe* on WLI, TXI, and NBI, and *ΔEe* under iodine staining on WLI and TXI; visibility grade under iodine staining assessed by endoscopists on TXI and WLI; changes in L*a*b* values after iodine staining in the ESCC and surrounding mucosa localities; and changes in L*a*b* values under iodine staining between the ESCC and surrounding mucosa. The visibility grade was evaluated under discussion by two endoscopists (Tsunetaka Kato and Ryoichiro Kobashi) for the same endoscopic images before *ΔE*e analysis. The visibility grade was defined as follows (Figure S1): Grade 1 when ESCC detection and border demarcation are difficult to perform, Grade 2 when ESCC detection is possible but border demarcation is difficult, Grade 3 when ESCC detection is possible but border demarcation is partially difficult, Grade 4 when ESCC detection is possible and border demarcation is generally possible, and Grade 5 when both ESCC detection and border demarcation are easy.

### Statistical analysis

Continuous values are reported as medians with 95% confidence intervals (CIs), and discrete values are reported as medians with ranges. The difference in median *ΔEe* between WLI, NBI, TXI, and WLI and TXI under iodine staining was subjected to a multiple comparison test (Bonferroni method). Lesion factors associated with increased *ΔEe* were tested using the Mann–Whitney U test. The visibility grade accessed by endoscopists was evaluated using the chi‐square test or m × n contingency table, and a comparison of L*a*b* values under iodine staining on WLI and TXI was analyzed using the Wilcoxon signed‐rank sum test. Pearson's correlation coefficient test was used to analyze the correlation of *ΔEe* under iodine staining on TXI and WLI. The correlation of *ΔEe* and the correlation of visibility grades under iodine staining on WLI and TXI were calculated using Spearman's rank correlation coefficient. The difference of *p*‐value < 0.005 for the subjected to a multiple comparison test (Bonferroni method) and *p*‐value < 0.05 otherwise was considered statistically significant, and statistically analyzed using the Statistical Package for the Social Sciences (version 21; IBM Corp).

## RESULTS

### Patients and lesion characteristics

Twenty lesions from the 20 patients who underwent ESD for ESCC during the study enrollment period were analyzed (Table 1). The median age of the patients was 71 years, and 90% were male. Lesion color was red, isochromatic, and white in 65%, 20%, and 15% of the cases, respectively, and lesion circumference less than 1/2 circumference was observed in 95% of the cases. All analyzed ESCCs were successfully treated with ESD and resected en bloc.

**TABLE 1:** Patient characteristics at baseline before endoscopic submucosal dissection of esophageal squamous cell carcinoma, and histopathological findings of resected specimens (*n* = 20).

Median age, (range), years	71 (60–80)
Sex, *n* (%)	
Male	18 (90)
Female	2 (10)
Lesion location, *n* (%)	
Upper thoracic esophagus	1 (5)
Middle thoracic esophagus	14 (70)
Lower thoracic esophagus	5 (25)
Macroscopic type, *n* (%)	
Slightly depressed	12 (60)
Flat	5 (25)
Slightly elevated	3 (15)
Preprocedural lesion depth estimation, *n* (%)	
Mucosa	60 (100)
Lesion circumference, *n* (%)	
<1/4	9 (45)
≥1/4–<1/2	10 (50)
≥1/2	1 (5)
Lesion color compared to the surrounding mucosa, *n* (%)	
Red	13 (65)
Isochromatic	4 (20)
White	3 (15)
Histopathological findings of resected specimens with ESD	
Lesion diameter, median (range), mm	14.5 (5–49)
Lesion depth, *n* (%)	
EP	6 (30)
LPM	10 (50)
MM	2 (10)
SM1	2 (10)
Lymphatic invasion, *n* (%)	1 (5)
Venous invasion, *n* (%)	0
Horizontal margin positive, *n* (%)	0
Vertical margin positive, *n* (%)	0

Abbreviations: EP, epithelium; ESD, endoscopic submucosal dissection; LPM, lamina propria mucosae; MM, muscularis mucosae; SM1, invasion depth <200µm from the muscularis mucosa.

### *ΔE*e in each image

**FIGURE 3:**
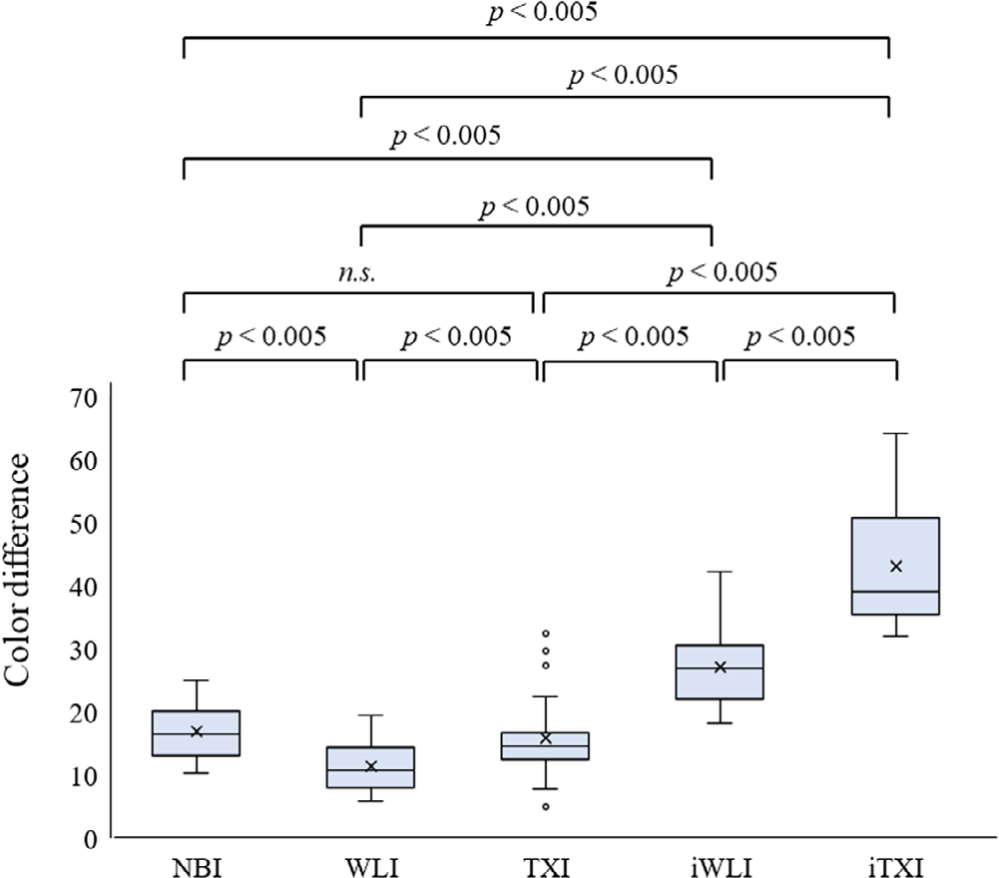
The color difference between the esophageal squamous cell carcinoma and surrounding mucosa (*ΔE*_e_). *ΔE*e in iTXI was greater than that in iWLI; *ΔE*e in iWLI was greater than that in WLI; and *ΔE*e in iTXI was greater than that in TXI. No significant difference in *ΔE*e was observed between TXI and NBI; however, TXI had a greater *ΔE*e than WLI. *ΔEe* in both iWLI and iTXI was greater than that in NBI. An adjusted two‐sided *p*‐value threshold of 0.005 was used using the Bonferroni method. *ΔE*e, the color difference between the esophageal squamous cell carcinoma and surrounding mucosa; TXI, texture, and color enhancement imaging; WLI, white light imaging; NBI, narrow‐band imaging; iTXI, TXI image under iodine staining; iWLI, WLI image under iodine staining; n.s., not significant

No difference in the median *ΔE*e was observed between TXI and NBI; however, the median *ΔE*e on both images was greater than that on WLI (14.53 vs. 16.42 vs. 10.71, respectively; *p* < 0.005 for both; Table 2 and Figure 3). The median *ΔE*e under iodine staining on WLI was greater than *ΔE*e on WLI (26.91 vs. 10.71, respectively; *p* < 0.005), and the median *ΔE*e on TXI under iodine staining was greater than *ΔE*e on TXI and NBI (39.20 vs. 14.53 vs. 16.42, respectively; *p* < 0.005 for both; Figure 3). A positive correlation of *ΔE*e under iodine staining was observed on TXI and WLI (correlation coefficient = 0.61, 95% CI: 0.23–0.83, *p* < 0.01), and *ΔE*e on TXI in each case was greater than the corresponding *ΔE*e under iodine staining on WLI (Figure S2). None of the lesion factors, including macroscopic type, lesion diameter, lesion depth, lesion circumference, or lesion color affected the *ΔE*e under iodine staining on TXI (Table 3).

**TABLE 2:** Median color difference value between esophageal squamous cell carcinoma and surrounding mucosa (*n* = 20).

	*ΔEe*, median (95% CI)
No staining	
WLI	10.71 (9.60–13.32)
NBI	16.42 (15.04–18.97)
TXI	14.53 (12.69–19.05)
With iodine staining	
iWLI	26.91 (24.27–30.24)
iTXI	39.20 (38.91–47.26)

Abbreviations: *ΔEe*, color difference between esophageal squamous cell carcinoma and surrounding mucosa; CI, confidence interval; iTXI, TXI image under iodine staining; iWLI, WLI image under iodine staining; NBI, narrow band imaging; TXI, texture and color enhancement imaging; WLI, white light imaging.

**TABLE 3:** Median color difference values in texture and color enhancement imaging with iodine staining according to lesion factors (*n* = 20).

	*ΔEe*, median (95% CI)	*p*‐value
Macroscopic type		
Slight depressed (*n* = 12)	39.20 (37.96–49.60)	0.64
Flat or slightly elevated (*n* = 8)	40.29 (35.93–48.15)	
Lesion diameter, mm		
< 15 (*n* = 10)	37.44 (34.92–46.67)	0.64
≥ 15 (*n* = 10)	47.61 (39.50–51.26)	
Lesion depth, *n* (%)		
EP to LPM (*n* = 16)	41.86 (39.40–48.54)	0.26
MM to SM1 (*n* = 4)	35.17 (28.86–50.23)	
Cancer circumference		
< 1/4 (*n* = 9)	38.22 (35.20–47.90)	0.52
≥ 1/4 (*n* = 11)	44.46 (38.65–50.03)	
Lesion color compared to the surrounding mucosa		
Red (*n* = 13)	36.67 (36.89–48.79)	0.66
Isochromatic/ white (*n* = 7)	43.91 (38.44–48.62)	

Abbreviations: *ΔEe*, color difference between esophageal squamous cell carcinoma and surrounding mucosa; CI, confidence interval; EP, epithelium; LPM, lamina propria mucosae; MM, muscularis mucosae; SM1, invasion depth <200 µm from the muscularis mucosa.

*p*‐Value was calculated by the Mann–Whitney U test.

### Visibility evaluation by endoscopists

In all lesions, the visibility grade evaluated under iodine staining on both TXI and WLI was≥4. However, the number of lesions with visibility grade 5 under iodine staining was 19 on TXI and 7 on WLI, with a significant difference (*p* < 0.01; Table 4). The visibility grades evaluated under iodine staining on TXI were significantly greater than those evaluated under iodine staining on WLI (Table 4). No difference in visibility grade distributions was observed among the evaluations on WLI, TXI, and NBI. A positive correlation was observed between *ΔEe* and subjective visibility scores by the endoscopist under iodine staining on a pooled analysis of WLI and TXI (correlation coefficient = 0.68, 95% CI: 0.35–0.77, *p* < 0.01; Figure S3).

**TABLE 4:** Visibility grade accessed by endoscopists (*n* = 20)

	Grade 1	Grade 2	Grade 3	Grade 4	Grade 5	*p*‐value
No staining						
WLI	3	7	8	2	0	0.14^*^
NBI	0	2	11	6	1	
TXI	1	8	7	4	0	
With iodine staining						
iWLI	0	0	0	13	7	<0.01^**^
iTXI	0	0	0	1	19	

Abbreviations: iTXI, TXI image under iodine staining; iWLI, WLI image under iodine staining; NBI, narrow band imaging; TXI, texture and color enhancement imaging; WLI, white light imaging.

Values are shown as *n*.

The visibility grade was defined as follows: Grade 5, both detection of the lesions and border demarcation are easy. Grade 4, the lesions can be detected, and border demarcation is generally possible. Grade 3, the lesions can be detected, but border demarcation is difficult in some parts of the lesion. Grade 2, the lesions can be detected, but border demarcation is difficult. Grade 1, both detection of the lesions and border demarcation are difficult.

* *p*‐Value was calculated by m × n contingency table.

** *p*‐Values were calculated by Chi‐square test.

### Analysis of L*a*b* values under iodine staining

**FIGURE 4:**
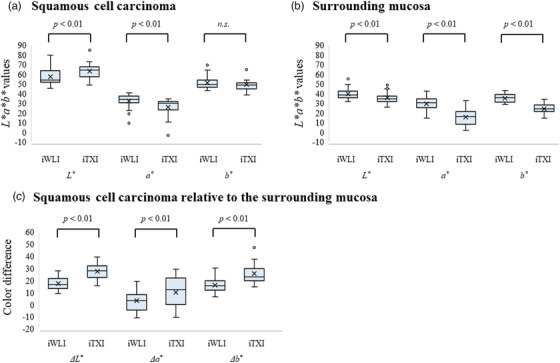
Component‐specific changes in ΔL*, Δa*, and Δb* in esophageal squamous cell carcinoma localization and surrounding mucosa localization, as well as between esophageal squamous cell carcinoma and surrounding mucosa, before and after iodine staining. L*a*b* values on WLI and TXI according to components in the esophageal squamous cell carcinoma (a) and surrounding mucosa (b) after iodine staining. After iodine staining, the L* component was significantly greater in the esophageal squamous cell carcinoma (ESCC) and significantly lower in the surrounding mucosa than that on WLI for TXI. The a* component was significantly lower in both the ESCC and surrounding mucosa than that on WLI for TXI. The b* component was significantly lower in the surrounding mucosa than that in WLI for TXI, but not in the ESCC. L*a*b* values of esophageal squamous cell carcinoma relative to the surrounding mucosa (c). All components of L*a*b* values for ESCC relative to the surrounding mucosa were significantly greater on iTXI than on iWLI. ESCC, esophageal squamous cell carcinoma; iTXI, TXI image under iodine staining; iWLI, WLI image under iodine staining; n.s., not significant

In the ESCC lesion, the median lightness component L* under iodine staining was significantly greater on TXI than on WLI (64.10 vs. 54.19, *p* < 0.01), and the median chromaticity component a* under iodine staining was significantly lesser on TXI than on WLI (29.79 vs. 33.90, respectively; *p* < 0.01), whereas the median chromaticity component b* under iodine staining did not significantly differ between TXI and WLI (48.60 vs. 49.12, respectively; *p* = 0.30; Figure 4a). In the surrounding mucosa, the median of all components of L*a*b* values under iodine staining were significantly lesser on TXI than on WLI (L*: 34.79 vs. 38.76, *p* < 0.01; a*: 16.33 vs. 30.56, *p* < 0.01; b*: 24.10 vs. 35.75*, p* < 0.01, respectively; Figure 4b). Furthermore, the median L*a*b* value under iodine staining of ESCC relative to the surrounding mucosa was significantly greater on TXI than on WLI for all components (ΔL*: 27.80 vs. 16.67, *p* < 0.01; Δa*: 12.53 vs. 3.19, *p* < 0.01; Δb*: 22.73 vs. 15.43, *p* < 0.01, respectively; Figure 4c).

## DISCUSSION

In this study, we evaluated the visibility of ESCC, mainly under iodine staining on WLI and TXI, using *ΔE*e as an objective parameter and the visibility grade assessed by endoscopists. The median *ΔE*e under iodine staining on TXI was significantly greater than that under iodine staining on WLI, and each *ΔE*e value under iodine staining on TXI was greater than the corresponding *ΔE*e value under iodine staining on WLI in all cases. No lesion factors that affect *ΔE*e were found. Furthermore, similar results were obtained in the subjective assessment of the visibility grade. The visibility of ESCC under iodine staining on TXI was correlated with the visibility under iodine staining on WLI and was better, and this was not affected by the lesion background.

TXI has been reported to be useful for the detection and border demarcation of neoplastic lesions in the stomach and colon.^18^, ^19^, ^20^, ^21^, ^22^, ^23^, ^28^, ^29^, ^30^, ^31^ These reports evaluated visibility using *ΔE*; however, had there been more pairs of lesions and surrounding mucosa in which *ΔE* was measured, they could have been classified as evaluating border demarcation. Abe et al. evaluated eight pairs of *ΔE* between early gastric cancer and surrounding mucosa and showed that *ΔE* increased under TXI observation, indicating the utility of TXI for border demarcation.^21^ Other studies have shown that TXI improves lesion visibility by measuring 1–4 pairs of *ΔE* between the lesion and surrounding mucosa or by using subjective visibility scores.^19,22,^ ^28^, ^29^, ^30^, ^31^ However, regarding TXI for esophageal lesions, only one ESCC study^24^ and only two studies on Barrett's esophagus^32^, ^33^ have been reported. Dobashi et al. evaluated the *ΔE*e in three pairs between the SCC and surrounding mucosa in the esophagus and pharynx and concluded that *ΔE*e on TXI Mode 1, TXI Mode 2, and NBI was significantly greater than that on WLI.^24^ Our present study evaluated the *ΔE*e derived from five pairs randomly selected from the entire ESCC, thereby allowing us to show the usefulness of TXI observation under iodine staining for ESCC border demarcation.

On TXI observation under iodine staining, the L* value between the ESCC and surrounding mucosa increased given that the brightness component L* in the ESCC was greater, whereas that in the surrounding mucosa was smaller, than that on WLI. Therefore, the combination of iodine staining and TXI made ESCC brighter and the surrounding mucosa darker, thereby improving visibility. Furthermore, compared to WLI, both a* and b* chromaticity components between the ESCC and surrounding mucosa on TXI increased, suggesting that the color enhancement function of TXI Mode1 also improved visibility. This study found that all Lab components were lower with TXI than with WLI under iodine staining for the surrounding mucosa and that the *ΔEe* of WLI and TXI under iodine staining was correlated. This suggests that the use of TXI may have the same effect as that of a high concentration of iodine staining.

Iodine solution for ESCC diagnosis is used in the range of 0.5%–2%, depending on the institution.^5^, ^16^, ^34^, ^35^, ^36^ The concentration of iodine solution should be as low as possible to prevent adverse events due to irritation.^35^ In a double‐blind randomized trial, Gotoda et al. showed that staining with 1% iodine solution had similar diagnostic accuracy to 2% iodine solution and caused less posterior chest pain.^16^ As long as the diagnostic performance is not decreased, a lower concentration of iodine solution is desirable. Although the concentration of iodine solution used in this study was 1%, the positive added visibility values observed here indicated that the use of TXI Mode1 may contribute to the border demarcation of ESCC even with a lower concentration of iodine solution.

Regarding the WLI, *ΔEe* values were greater with iodine staining than without, in all cases. However, only one lesion with an unchanged visibility grade on WLI under iodine staining as assessed by endoscopists was observed. Given that the lesion was stained with iodine, it was difficult to evaluate the contrast with the surrounding mucosa. Considering that all images were taken within 1 min after iodine staining, the pink sign and its effect on *ΔEe* remained unknown. Histologically, this lesion was considered to be caused by the high glycogen content of the mucosa in ESCC, which stained ESCC with iodine. Moreover, the increase in visibility grade from 4 to 5 indicates the borderline between the lesion and surrounding mucosa is clearer, which reduced the endoscopist's concern during ESD marking. Although we could not objectively evaluate the visibility grade and the stress induced by marking during ESD, the endoscopists were certainly concerned when the visibility grade was 4 points.

This study has several limitations. First, this was a single‐center, retrospective study involving consecutive patients with a small sample size. Second, *ΔE*e was evaluated on WLI, TXI, and NBI, and the distance and angle to the endoscope were not always the same. An image selection bias may also have been present given that the evaluation was based on the limited number of images obtained. Third, we did not evaluate *ΔEe* under iodine staining on NBI because it is uncommon to use iodine on NBI for border demarcation between the ESCC and surrounding mucosa. Finally, this study used only a 1% concentration of iodine staining and did not use lower concentrations of iodine.

In conclusion, under 1% iodine staining, the positive effects of TXI on ESCC border demarcation compared with WLI were demonstrated. Further prospective studies using TXI and lower‐concentration iodine solutions are required.

## CONFLICT OF INTEREST STATEMENT

None.

## ETHICS STATEMENT

This study was approved by the Ethics Committee of Fukushima Medical University (No. 2020–146)

## Supporting information


**FIGURE S1** Typical images for visibility grade evaluation.
Grade 1, lesion detection and border demarcation are difficult (a). Grade 2, lesions can be detected, but border demarcation is difficult (b). Grade 3, lesions can be detected, but border demarcation is difficult in certain parts of the lesion (c). Grade 4, the lesions can be detected, and border demarcation is generally possible (d, e). Grade 5, lesion detection and border demarcation are easy (f). Figure S1a–d depicts the endoscopic images obtained through white light imaging, whereas, Figure S1e,f depicts the endoscopic images obtained through white light imaging under iodine staining. The yellow arrow in Figure S1b indicates a lesion.

**FIGURE S2** Correlation between *ΔE*e in iTXI and *ΔE*e in iWLI.
A positive correlation was observed between *ΔE*e in iTXI and that in iWLI (correlation coefficient [R] = 0.61, 95% confidence interval: 0.23–0.83, *p* < 0.01), and *ΔEe* was greater in iTXI than in iWLI in all cases.
*ΔE*e, the color difference between the esophageal squamous cell carcinoma and surrounding mucosa; iTXI, TXI image under iodine staining; iWLI, WLI image under iodine staining.

**FIGURE S3** Correlation between the visibility grade and *ΔEe* containing iWLI and iTXI.
A positive correlation was observed between the visibility grade and *ΔEe* containing iWLI and iTXI (correlation coefficient [ρ], 0.68; 95% confidence interval: 0.35–0.77; *p* < 0.01).
*ΔEe*, the color difference between the esophageal squamous cell carcinoma and surrounding mucosa; iTXI, TXI image under iodine staining; iWLI, WLI image under iodine staining.

## Data Availability

We would like to provide the data as needed.
