# Supplementary figures and images for: Visibility of esophageal squamous cell carcinoma under iodine staining on texture and color enhancement imaging
Source: DEN Open. 2024 May 8;5(1):e370. doi: 10.1002/deo2.370 (PMC11079435; doi:10.1002/deo2.370)

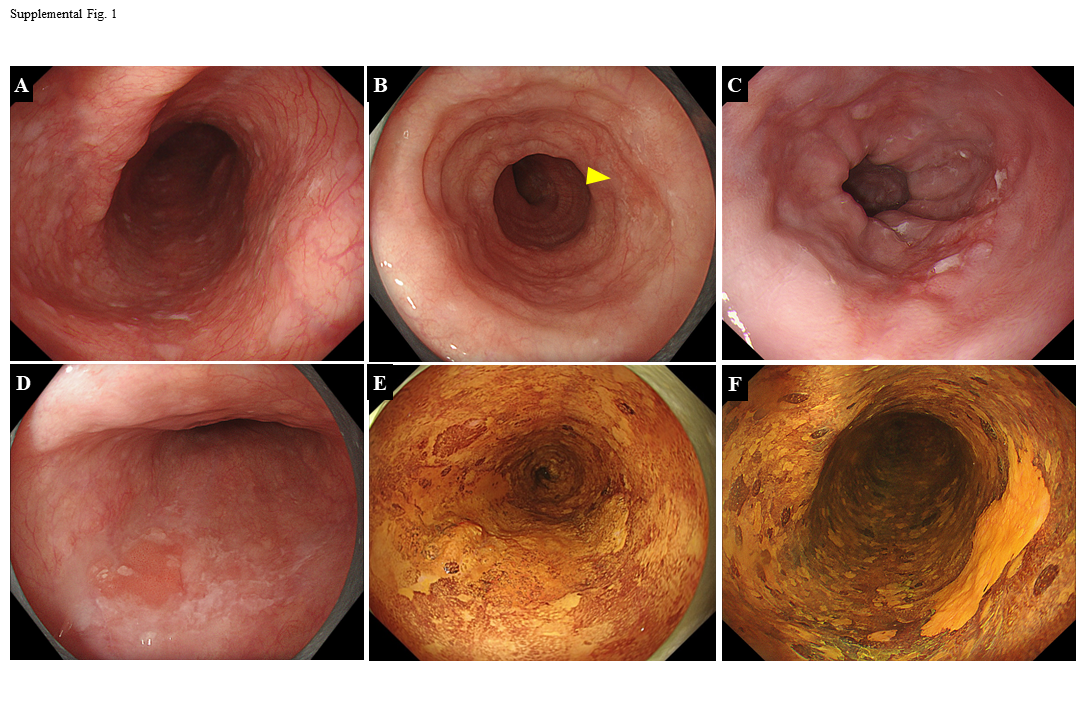

Supplement: Supplementary file 1 — FIGURE S1 Typical images for visibility grade evaluation. Grade 1, lesion detection and border demarcation are difficult (a). Grade 2, lesions can be detected, but border demarcation is difficult (b). Grade 3, lesions can be detected, but border demarcation is difficult in certain parts of the lesion (c). Grade 4, the lesions can be detected, and border demarcation is generally possible (d, e). Grade 5, lesion detection and border demarcation are easy (f). Figure S1a–d depicts the endoscopic images obtained through white light imaging, whereas, Figure S1e,f depicts the endoscopic images obtained through white light imaging under iodine staining. The yellow arrow in Figure S1b indicates a lesion. [file DEO2-5-e370-s003.tif]

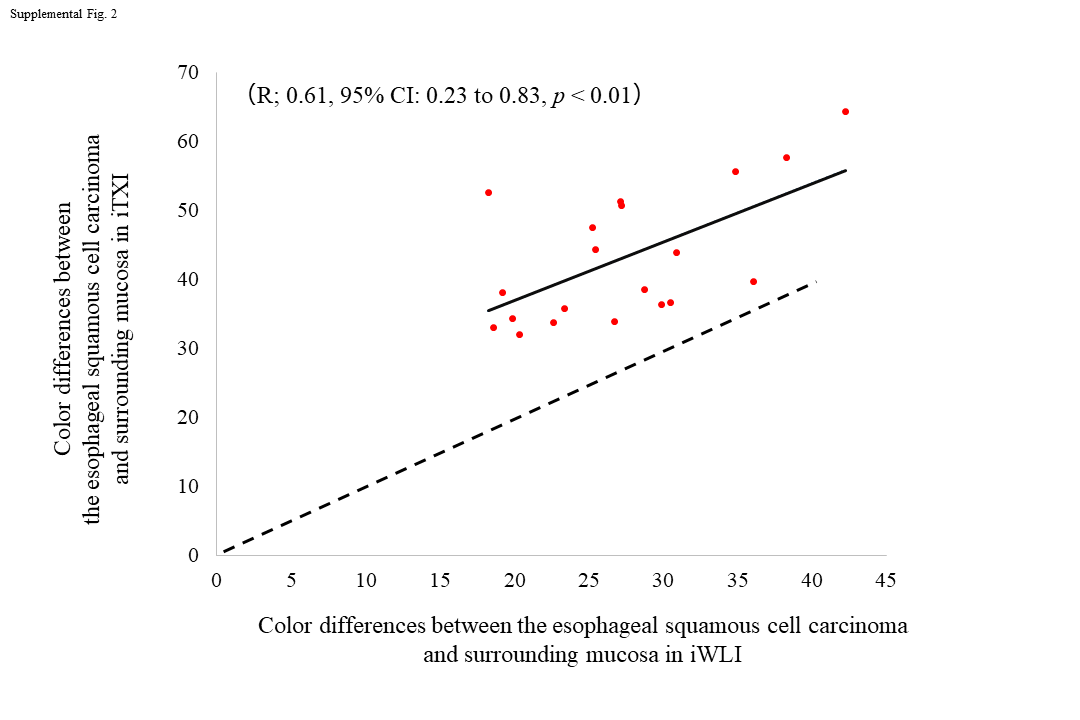

Supplement: Supplementary file 2 — FIGURE S2 Correlation between ΔEe in iTXI and ΔEe in iWLI. A positive correlation was observed between ΔEe in iTXI and that in iWLI (correlation coefficient [R] = 0.61, 95% confidence interval: 0.23–0.83, p < 0.01), and ΔEe was greater in iTXI than in iWLI in all cases. ΔEe, the color difference between the esophageal squamous cell carcinoma and surrounding mucosa; iTXI, TXI image under iodine staining; iWLI, WLI image under iodine staining. [file DEO2-5-e370-s002.TIF]

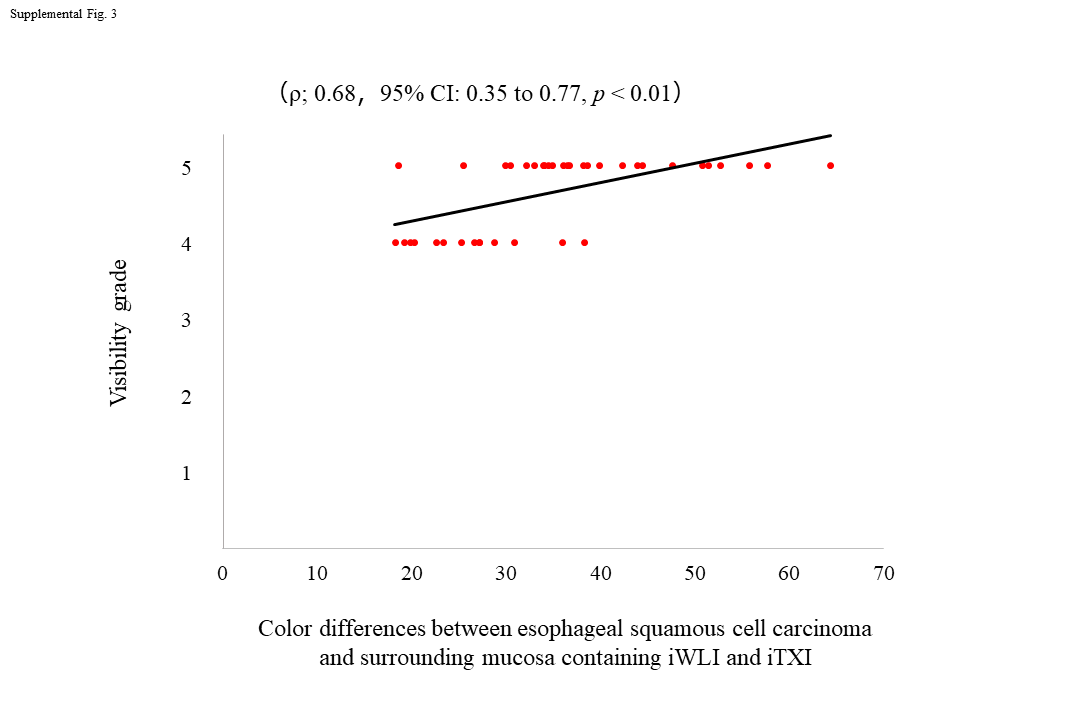

Supplement: Supplementary file 3 — FIGURE S3 Correlation between the visibility grade and ΔEe containing iWLI and iTXI. A positive correlation was observed between the visibility grade and ΔEe containing iWLI and iTXI (correlation coefficient [ρ], 0.68; 95% confidence interval: 0.35–0.77; p < 0.01). ΔEe, the color difference between the esophageal squamous cell carcinoma and surrounding mucosa; iTXI, TXI image under iodine staining; iWLI, WLI image under iodine staining. [file DEO2-5-e370-s001.TIF]
